# Dr. Magennis, on Epilepsy

**Published:** 1800-11

**Authors:** J. Magennis

**Affiliations:** Royal Hospital, Plymouth


### *To the Editors of the Medical and Phyſical Journal.*

Gentlemen,

A Faithful and unbiaſſed hiſtory of facts, whether favourable or otherwiſe, as they occur in the treatment of every diſeaſe, and an impartial narrative of the action of every new remedy as it appears to affect the living ſyſtem, would tend to elucidate many obſcure points in medicine, and greatly enlarge the ſphere of our knowledge; but it is to be lamented, that in the ardour of a new diſcovery, ſimple alleviation of urgent ſymptoms is too frequently magnified into a perfect cure, and a real or apparently ſucceſsful caſe or two, at once ſtamps the remedy with the character of infallibility. Hence, the frequent diſappointments experienced by others; and hence, the total rejection of a valuable medicine is ſometimes the conſequence of theſe exaggerated and hyperbolic praiſes.

Men are in general fond of the marvelous, and they are anxious to excite it in others; ſelf-love, and a fear of public cenſure, often induce them to conceal the unſucceſsful part of their practice, whilſt they relate with ſtudied care and attention the fortunate caſes only. Not ſatisfied with this diſingenuous concealment, it is much to be feared, that many are to be found in the profeſſion, who detail with circumſtantial minuteneſs, caſes which, probably, never had exiſtence, except in their own imaginations; or, having actually occurred, who warp facts, ſymptoms, and circumſtances, to force a coincidence with ſome adopted opinions, or for the purpoſe of ſupporting ſome preconceived and favourite theory. This weak, and we may juſtly add, criminal conduct, is productive of ſerious evils, as it not only miſleads the credulous and inexperienced practitioner, but tends to render error ſyſtematically permanent.

I have been led into theſe reflections from comparing the ſucceſsful practice of others, in the treatment of Epilepſy, with my own, which to my no ſmall regret and mortification, has hitherto proved uniformly unfavourable. At this I have reaſon to be the more ſurprized, as I had employed preciſely the

ſame fame remedies which were given with fuch very diffimilar refults by many of your correfpondents; and what adds to the fingularity of my attempts is, that I pufhed the dofes of medicines beyond all previous example or precedent, fo far at leaft as I am acquainted. For inftance, that of the nitrate of filver, mentioned by P. Mudie of Arborth, Dr. Cappe of York, and Dr. Sims, with fuch commendations. I have adminiftered it in chronic and recent cafes of epilepfy, to the quantity of twelve grains in a day, without any one perceivable effect; that is, four grains at a dofe given ter in die, having commenced with dofes of half a grain, which were gradually and daily increafed.

Again, the cupr. ammon. fo celebrated in the hands of others, has proved like the argent. nitrat. equally inert and unfuccefsful in mine. It was exhibited in fmall dofes at firft, and continued till the quantity amounted to twenty-four grains a day; and this increafed quantity was perfevered in for fifteen days, but without the fmalleft advantage or effect.

Eleven epileptic patients, who came under my care nearly about the fame time, were claffed and treated in the following order:

Three patients took the argent. nitrat. gr. iv. ter in die.

Two took the cupr. ammon. the largeft dofe, gr. viij ter die.

Two, pulv. cinchon. ℈iv. cupr. vitriol. gr. iifs. M. quart. quaq. hor. That is, one ounce of bark, and fifteen grains of the cupr. vitr. daily.

Two took the pulv. valer. filv. ʒj. quart. quaq. hor. or one ounce daily.

Two took the arfen. alb. to the quantity of one grain and a half daily.

It is to be recollected, that the active medicines were adminiftered in very reduced dofes at the commencement, and gradually increafed till the quantity amounted to what is above fpecified. One tenth of a grain of arfenic was at firft exhibited every four hours, and augmented by regular gradation to one fourth of a grain; but beyond this it could not be carried. Half a grain of the cupr. vitriol. given every four hours, was the dofe commenced with, and increafed to two grains and a half. Neither this or the preceding medicine could be pufhed farther, as the ftomach became affected; and even the above quantities frequently occafioned naufea and vomitings, efpecially when given on an empty ftomach, the ficknefs always recurring in the morning. This plan was perfevered in for fix weeks, and the refult was in all uniformly the fame; that is, no amendment whatever took place.

The

The argent. nitrat. was again tried on a patient lately under my care at the Royal Hoſpital, of which he took ten grains daily, with reſults preciſely ſimilar to thoſe already mentioned. The paroxyſms in four of them obſerved regular periods like intermittents of the tertian and quartan type, and thoſe were ordered the cinchon. cupr. vitriol. arſen. alb. and occaſionally large doſes of opium. The recurrence of paroxyſms in ſix of them, was uſually more frequent and violent during the full and change of the moon; and one man in particular had rarely more than one fit, except at theſe periods, when he was ſure to have a regular daily attack. Two others, who were ſubject to naſal hæmorrhagies and a tempory inflammation of the tunica conjunctiva of the eye, during the attack, had their heads ſhaved, bliſters applied to the ſutures, and leeches to the temples.

I obſerved in theſe patients, and in moſt others who have long laboured under this untoward diſeaſe, a dulneſs of apprehenſion, a particular ſtare and vacuum of countenance, a dilated pupil, and an inability of the iris to contract on the admiſſion of light, accompanied with ſtupor and a general irritability of the muſcular fibre. This torpor extends to the ſtomach and inteſtinal canal, as thoſe people ſubject to the diſorder uſually require the moſt active cathartics and emetics to excite the primæ viæ into action.

I truſt that theſe remarks will not be conſidered as reflecting on the ability or veracity of the reſpectable names already mentioned, nothing is indeed farther from my intention, their characters and talents are too well known to the medical world to need even this explanation; but every practitioner, however ſkilful and enlightened, is at times liable to error, eſpecially when drawing concluſions from premiſes new and imperfectly underſtood. That the timid and feeble practice of theſe gentlemen in the treatment of epilepſy ſhould have been ſucceſsful, whilſt the bold and deciſive, but at the ſame time cautious practice of another, had entirely failed, is indeed to be wondered at; and will induce us to infer, that appearances with reſpect to the effects of the remedy, had, ſome how or other, certainly deceived them; for we cannot ſuppoſe, that medicines which had been exhibited in ſuch large doſes, whoſe uſe had been perſevered in for a length of time, and which not only failed in performing a cure, but proved perfectly inert on every occaſion, ſhould have ſucceeded, when given in ſuch minute and divided portions.

Our knowledge of the cauſes and cure of this frightful malady is, indeed, extremely limited, and little or no dependance can be placed on all the boaſted medicines hitherto diſcovered vered and recommended for it; but these difficulties ought not to suspend the efforts, or deaden the ardour of the humane and enlightened physician, they ought rather to stimulate him to greater exertions in the pursuit of new remedies, or in differently combining and applying those already known, in order to combat successfully a disease, which must at present be reckoned in the number of the opprobria medicinæ.

If these observations be deemed worthy a place in your valuable Journal, they are much at your service.—In my next Communication, I propose laying before you the farther results of my experiments on the Digitalis Purpurea, in the treatment and cure of Phthisis Pulmonalis. I am,

GENTLEMEN,

Your obedient humble servant,

*Royal Hospital, Plymouth,* J. MAGENNIS, M.D.

